# MTR-Viewer: identifying regions within genes under purifying selection

**DOI:** 10.1093/nar/gkz457

**Published:** 2019-06-06

**Authors:** Michael Silk, Slavé Petrovski, David B Ascher

**Affiliations:** 1 Department of Biochemistry and Molecular Biology, University of Melbourne, Melbourne, VIC 3052, Australia; 2 ACRF Facility for Innovative Cancer Drug Discovery, Bio21 Institute, University of Melbourne, Melbourne, VIC 3052, Australia; 3 Structural Biology and Bioinformatics, Baker Heart and Diabetes Institute, Melbourne, VIC 3004, Australia; 4 Centre for Genomics Research, Precision Medicine and Genomics, IMED Biotech Unit, AstraZeneca, Cambridge, UK; 5 Department of Medicine, The University of Melbourne, Austin Health and Royal Melbourne Hospital, Melbourne, VIC 3050, Australia; 6 Department of Biochemistry, University of Cambridge; Cambridge CB2 1GA, UK

## Abstract

Advances in genomic sequencing have enormous potential to revolutionize personalized medicine, however distinguishing disease-causing from benign variants remains a challenge. The increasing number of human genome and exome sequences available has revealed areas where unfavourable variation is removed through purifying selection. Here, we present the MTR-Viewer, a web-server enabling easy visualization at the gene or variant level of the Missense Tolerance Ratio (MTR), a measure of regional intolerance to missense variation calculated using variation from 240 000 exome and genome sequences. The MTR-Viewer enables exploration of MTR calculations, using different sliding windows, for over 18 000 human protein-coding genes and 85 000 alternative transcripts. Users can also view MTR scores calculated for specific ethnicities, to enable easy exploration of regions that may be under different selective pressure. The spatial distribution of population and known disease variants is also displayed on the protein's domain structure. Intolerant regions were found to be highly enriched for ClinVar pathogenic and COSMIC somatic missense variants (Mann–Whitney U test *P* < 2.2 × 10^−16^). As the MTR is not biased by known domains and protein features, it can highlight functionally important regions within genes overlooked or inaccessible by traditional methods. MTR-Viewer is freely available via a user friendly web-server at http://biosig.unimelb.edu.au/mtr-viewer/.

## INTRODUCTION

Exome sequencing is becoming a routine tool to guide personalized medicine of genetic diseases (1,2), including in the diagnosis of many Mendelian genetic diseases and to guide cancer treatment decisions (3). While this has lead to a growing library of variants with evidence of pathogenicity (4–6), many variants in a patient's exome remain of uncertain significance. *In silico* predictors of deleteriousness are used to prioritize likely candidate variants, but it remains a major challenge to discriminate pathogenic from benign variants (7).

Large exome (8) and genome (9) sequencing projects have yielded references of variation across the human genome providing the means to measure patterns of variability within genes (10,11). It has been demonstrated previously that measuring depletion of standing variation within genes can be used to identify novel disease-associated genes (10,11). With the current sample sizes of sequenced individuals, we can begin to measure depletion of variation at a regional level within these genes.

We have shown that the Missense Tolerance Ratio (MTR), a measure of regional intolerance to missense variation, can capture this regional level information (12). The MTR is a direct measure of purifying selection of missense variation within a gene, calculated as a ratio between the observed proportion of missense variants compared to an expected proportion, estimated under the assumption of no selection occurring on that sequence context. A sliding window summation is used to provide accurate regional measurements. We have previously shown that regions measured as intolerant to missense variation are significantly enriched for pathogenic missense variants in epilepsy genes (12).

We introduce the MTR-Viewer, a web-server for evaluating missense variant deleteriousness by examining its surrounding regional intolerance. Missense variants that exist within regions that are measured as being intolerant regions are more likely to be pathogenic. The MTR-Viewer provides an easy-to-use interface for viewing a selected gene/transcript MTR estimates, also supporting ethnicity-based differences in purifying selection as well as the ability to query individual variants, including via an API, and view disease and background variants on the protein domain structure (http://biosig.unimelb.edu.au/mtr-viewer).

## MATERIALS AND METHODS

### Data sets

Population variation was sourced from gnomAD (8), the DiscovEHR dataset (13) and the UK Biobank (14), collaborative efforts to aggregate human exome and genome sequences. The amalgamated datasets from a total of 240 000 exome and genome sequences were filtered for only single-point variation with a quality control ‘PASS’ flag, as previously described (12).

Gene and protein sequences were acquired from the Ensembl database (v95) (15) using the R Bioconductor biomaRt package (16). Transcripts were only used where they contained at least one single-point variant in gnomAD and had non-ambiguous sequences. Ensembl transcript ID’s were queried for their matching HGNC gene symbols (17) and Refseq transcript ID’s (18) using the biomaRt package.

The observed proportion of missense variation was compared to an expected proportion of missense variation calculated under the assumption of neutrality where no positive/negative selection is occurring. All possible single-point mutations within all gene transcripts were labelled by the Variant Effect Predictor (Release 95) (15) as either missense or synonymous.

For validation purposes, the MTR scores were also calculated in the absence of the DiscovEHR dataset. DiscovEHR missense variants not reported in gnomAD or the UK Biobank, and thus independent of the formulation of the MTR, were used as a control set of neutral variants.

For validation, ClinVar (19) missense variants were retrieved from the NCBI FTP database at ftp://ftp.ncbi.nlm.nih.gov/pub/clinvar/ and subset to pathogenic / likely pathogenic and benign / likely benign variants with no conflicting evidence.

For validation, COSMIC (20) missense variants were retrieved from their website at https://cancer.sanger.ac.uk/cosmic/download and filtered for confirmed somatic missense variants.

For further validation, the MTR scores were examined using the FATHMM inherited disease variant dataset and FATHMM cancer-associated missense variants dataset (21). These were compared to the results from the MPC (V2), a prediction of missense variant deleteriousness combining functional and regional missense intolerance information, downloaded from ftp://ftp.broadinstitute.org/pub/ExAC_release/release1/regional_missense_constraint/.

### Calculation of the missense tolerance ratio

The proportion of missense variants to synonymous variants was calculated for both the observed variation in gnomAD and the expected variation under neutrality using the annotations from all possible variants in a given transcript, as previously described (12). This was calculated over each Ensembl transcript using a sliding window of 21-, 31- and 41-codons. While using smaller window sizes can provide finer resolution, they can suffer from jitter caused by limited information per window. For this reason we recommend to use 31-codon as the default (12).

For a given window $W_i^{H,J}$ and with selected window size w,

(1) \begin{equation*}\begin{array}{@{}*{1}{l}@{}} {{\rm{where}}\,{{i}} = {\rm{amino}}\,{\rm{acid}}\,{\rm{position}}}\\ {{{H}} = {\rm{max}}\left( {1,\,{{i}}-\left( {{{w}} - 1} \right)/2} \right)}\\ {{{J}} = {\rm{min}}\left( {{\rm{transcript}}\,{\rm{length,}}\,{{i}} + \left( {{{w}} - 1} \right)/2} \right){\rm{,}}} \end{array}\end{equation*}

Within each window (Equation 1), the missense and synonymous variants are each summed at each amino acid position *y_i_* for both the observed and expected datasets (Equation 2).

(2) \begin{equation*}{y_i} = \sum\limits_{{x_m} \in W_i^{H,J}} {{x_m}} \end{equation*}

\begin{eqnarray*}\forall x &\in& \{{\rm missense}\_{\rm obs},\ {\rm synonymous}\_{\rm obs},\nonumber\\ && {\rm missense}\_{\rm exp},{\rm synonymous}\_{\rm exp}\}\end{eqnarray*}

Thus for each amino acid position, the MTR is calculated as follows.

(3) \begin{equation*}{\rm MT}{R_i} = \ \frac{{{\rm missense}\_{{\rm obs}_i}\ /\ \left( {{\rm missense}\_{{\rm obs}_i}\ + \ {\rm synonymous}\_{{\rm obs}_i}} \right)}}{{{\rm missense}\_{{\rm exp}_i}\ /\ \left( {{\rm missense}\_{{\rm exp}_i}\ + \ {\rm synonymous}\_{{\rm exp}_i}} \right)}}\end{equation*}

### FDR-adjusted binomial exact test

To identify significantly intolerant regions, an exact binomial test was performed at each residue position to test whether the regional observed proportion of missense variants significantly deviates from the expected proportion.

(4) \begin{equation*}{{P(X) = }}\frac{{n!}}{{(n - x)!x!}}\,{(p)^x}\,{(q)^{n - x}}\end{equation*}

\begin{equation*}\begin{array}{@{}*{1}{l}@{}} {{\rm{where}}\,n = {\rm{missense}}\_{\rm{obs}} + {\rm{synonymous}}\_{\rm{obs}}}\\ {{{x}} = {\rm{missense}}\_{\rm obs}}\\ {{{p}} = {\rm{missense}}\_{\rm{exp}}/({\rm{missense}}\_{\rm{exp}} + {\rm{synonymous}}\_{\rm{exp}})}\\ {{{q}} = 1 - {{p}}} \end{array}\end{equation*}

The exome-wide binomial exact test was then adjusted for False Discovery Rate (FDR) using the Benjamini–Hochberg method (22,23). FDR <0.1 was selected through empirical observation as accurately identifying intolerant regions.

## WEBSERVER

We have implemented the MTR-Viewer as a user-friendly and freely available web-server (http://biosig.unimelb.edu.au/mtr-viewer/). The webserver was developed using Python Flask (v1.0.2), formatted using Bootstrap (v4.1.3) with data stored using PostgreSQL 10.5. The Pfam API (24) is used to provide graphical domain representations for the accompanying Lollipop plots (25), obtained by translating the HGNC gene symbols (17) to UniProt accession numbers using the UniProt REST API (26). The web application is hosted on an Apache2 web-server running Ubuntu 16.04. MTR calculations were performed in R and plotting within the web application is performed using Python Bokeh (v1.0.1) (27).

### Input

The MTR-Viewer can be used in two different ways: either as a gene transcript viewer for MTR estimates across the entire protein-coding sequence or to query specific missense variants for the MTR scores at their position.

The gene viewer query page (Supplementary Figure S1) allows a user to input a specific HGNC gene symbol, which will default to our canonical-selected transcript, or to directly enter an Ensembl transcript ID or Refseq transcript ID. Names are not case sensitive.

The variant query page features a text box for users to input one or multiple missense variants on separate lines in formats chromosome-position-reference-alternative allele, chromosome-position or transcript-protein position. Positions are based on the GRCh37 reference genome. A query can also be performed using an API at http://biosig.unimelb.edu.au/mtr-viewer/api?q=<query> or as a CSV file upload.

Users may also search for a gene or transcript using an input box in the navigation bar on the results page, which will assume a gene is being queried unless formatted as a variant using delimiters.

### Output

**Figure 1.**
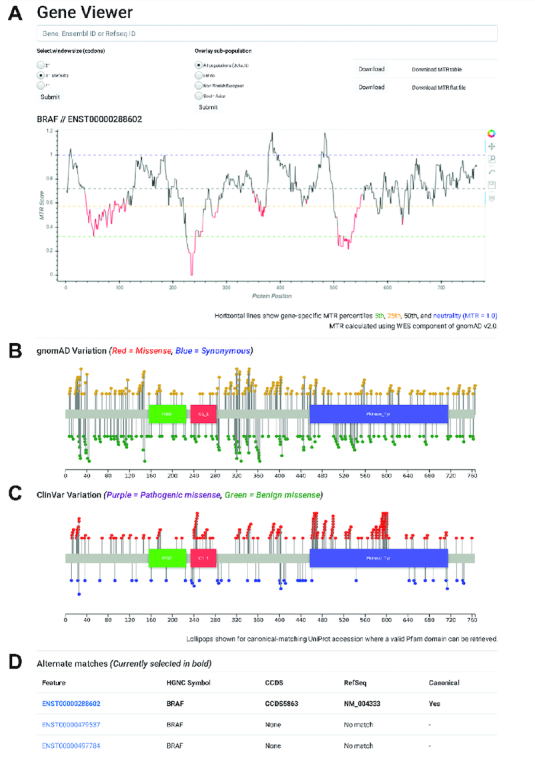
MTR-Viewer gene query results page. (**A**) A line graph displays the MTR distribution for example gene *BRAF* with regions in red indicating observed variation differs significantly from neutrality. (**B**) Lollipop plots show the underlying gnomAD missense and synonymous variation and (**C**) ClinVar known pathogenic and known benign variants for the gene. (**D**) Alternate transcripts are displayed below with matching RefSeq transcript ID’s.

The gene viewer results page (Figure 1) displays the MTR scores across the gene transcript as a line-graph. Line sections are coloured red where the FDR-adjusted binomial exact test <0.1, quantifying MTR deviation from neutrality (MTR = 1). Window sizes of 21 codons, 31 codons (default) and 41 codons can be displayed. Ethnicity-specific MTR estimates, calculated by filtering observed variation by ethnicity, can be overlaid. These are available only for ethnicities with over 15 000 exomes and for window sizes of 31 and 41 codons to account for the smaller sample size. Currently, this includes European Non-Finnish, Latino and South Asian populations. Hovering over the MTR line displays the amino acid position and corresponding MTR estimate. Buttons are available to drag-to-zoom, pan and download the table of raw data for the current transcript in flat file form (MTR estimates for individual genomic variants) or as an MTR table (MTR scores for amino acid positions) (see Figure 1).

Lollipop plots are also shown for the canonical transcript of the selected gene if a matching Pfam graphical representation is available (Figure 1). A lollipop plot is displayed to show the underlying distribution of gnomAD missense (yellow) and synonymous (green) variation and, if the gene is a ClinVar pathogenic gene, a second lollipop plot showing ClinVar annotated pathogenic (red) and benign (blue) variants.

The variant query results page (Supplementary Figure S2) displays a table of the input variants with their corresponding MTR estimates for all Ensembl gene transcripts (v95) that the variant is contained in. Variants with no match are reported in the results table. The view button will redirect the user to the gene viewer for that transcript and label the variant on the MTR line graph.

## VALIDATION

To further validate the utility of the MTR scores to differentiate pathogenic variants, we examined their distribution across the ClinVar pathogenic missense variant and Catalogue Of Somatic Mutations In Cancer (COSMIC) datasets.

**Figure 2.**
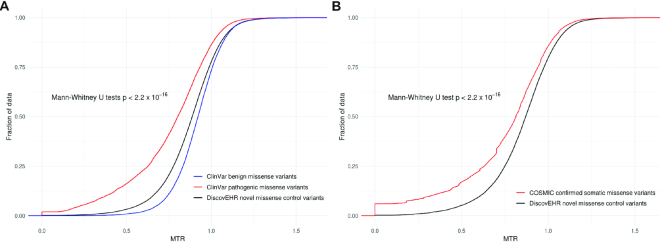
Distribution of MTR scores for known disease variants compared to background. (**A**) Cumulative distribution of MTR scores for ClinVar pathogenic variants (red), ClinVar benign variants (blue) and DiscovEHR novel missense control variants (black). (**B**) Cumulative distribution of MTR scores for COSMIC somatic missense variants (red) compared with DiscovEHR novel missense control variants (black).

The MTR scores of unique ClinVar pathogenic-assigned missense variants (*n* = 29 330, Average MTR = 0.77, MTR Standard Deviation = 0.24) were compared to the MTR scores of unique ClinVar benign-assigned missense variants (*n* = 18 582, Average MTR = 0.92, MTR Standard Deviation = 0.14) (Figure 2A). In addition, the pathogenic-assigned variants were also compared to the MTR scores from a novel set of missense variants not observed in gnomAD from the DiscovEHR reference cohort, and filtered to those within ClinVar genes (*n* = 195 735, average MTR = 0.87, MTR Standard Deviation = 0.18). ClinVar pathogenic-assigned variants were significantly more likely to occur in MTR missense depleted regions than the ClinVar benign variants or the novel population-based DiscovEHR missense variants (Mann–Whitney U test *P* values of < 2.2 × 10^−16^).

A comparison of confirmed somatic COSMIC variants (*n* = 231 724, average MTR = 0.74, MTR Standard Deviation = 0.28) to DiscovEHR population variation within COSMIC genes (*n* = 47 589, Average MTR = 0.85, MTR Standard Deviation = 0.19) was also performed to identify whether there is significant enrichment of cancer-ascertained somatic mutations within intolerant regions (Figure 2B). COSMIC variants were found to be significantly more likely to occur in intolerant regions (Mann–Whitney U test, *P* < 2.2 × 10^−16^).

The discriminatory power of the MTR scores was also assessed using the FATHMM SwissProt/TrEMBL training dataset and the FATHMM cancer-associated training dataset. When we evaluate missense variants with MTR scores less than 0.25 or 0.5, we found that 2.0% and 8.6% respectively of disease causing missense variants, but only 0.1% and 0.9% respectively from neutral variants reside in these regions (odds ratio [OR] = 13.76; Fisher's exact test *P* < 2.2 × 10^−16^, odds ratio [OR] = 10.11; Fisher's exact test *P* < 2.2 × 10^−16^). Similarly, we found that 2.1% and 9.7% of cancer asociated missense variants, but only 0.3% and 1.7% from neutral variants, have MTR less than 0.25 or 0.5 respectively (odds ratio [OR] = 6.49; Fisher's exact test *P* < 2.2 × 10^−16^, odds ratio [OR] = 6.36; Fisher's exact test *P* < 2.2 × 10^−16^). This showed that low-MTR scored regions are highly enriched for pathogenic variation.

We empirically selected FDR <0.1 to define regions with a significantly different proportion of observed missense variants. 10.5% of the FATHMM disease-associated variants and 9.6% of the cancer-associated variants are found in these regions, compared with 2.4% and 3.2% neutral variants, showing a significant enrichment of disease-associated variation in both datasets (odds ratio [OR] = 4.69; Fisher's exact test *P* < 2.2 × 10^−16^, odds ratio [OR] = 3.23; Fisher's exact test *P* < 2.2 × 10^−16^).

While the MTR is solely a measure of missense depletion, using the FATHMM training datasets, it was compared to the trained predictors MPC and PolyPhen-2, which utilize functional information (Supplementary Tables S1 and S2). The MTR had the highest Matthew's correlation coefficient over the FATHMM cancer-associated dataset, and was comparable to the MPC scores over the FATHMM disease-associated using the authors' defined cut-off of MPC >2.

## CONCLUSION

Here we present the MTR-Viewer, a web-server to explore regional intolerance to missense variation across human protein-coding genes from 240 000 exome and genome sequences. By providing a measure and visualization of purifying selection occurring within a given gene transcript, patient-ascertained variants can be better prioritized based on whether they reside in intolerant regions (12). The MTR-Viewer is freely available as a user-friendly web server at http://biosig.unimelb.edu.au/mtr-viewer/.

## Supplementary Material

gkz457_Supplemental_File
