# Supplementary material for: MTR-Viewer: identifying regions within genes under purifying selection
Source: Nucleic Acids Res. 2019 Jun 6;47(W1):W121–6. doi: 10.1093/nar/gkz457 (PMC6602522; doi:10.1093/nar/gkz457)
Supplement: gkz457_Supplemental_File [file gkz457_supplemental_file.docx]

# **SUPPLEMENTARY MATERIAL**

**MTR-Viewer: Identifying regions within genes under purifying selection**

Michael Silk^1,2,3^, Slavé Petrovski^4,5^, David B. Ascher^1,2,3,6,^*

^1^Department of Biochemistry and Molecular Biology, University of Melbourne; VIC, 3052, Australia

^2^ACRF Facility for Innovative Cancer Drug Discovery, Bio21 Institute, University of Melbourne; VIC, 3052, Australia

^3^Structural Biology and Bioinformatics, Baker Heart and Diabetes Institute, Melbourne, VIC, 3004, Australia

^4^Centre for Genomics Research, Precision Medicine and Genomics, IMED Biotech Unit, AstraZeneca, Cambridge, UK

^5^Department of Medicine, The University of Melbourne, Austin Health and Royal Melbourne Hospital, Melbourne, VIC, 3050, Australia

^6^Department of Biochemistry, University of Cambridge; Cambridge, UK

*To whom correspondence should be addressed D.B.A. Tel: +61 90354794; Email: [david.ascher@unimelb.edu.au](mailto:david.ascher@unimelb.edu.au) or [da382@cam.ac.uk](mailto:da382@cam.ac.uk).

**Table S1 – Performance of MTR, MPC and Polyphen-2 for distinguishing between disease-causing and neutral variants.**

| **Metric** | **TP** | **FP** | **TN** | **FN** | **Accuracy** | **Sensitivity** | **Specificity** | **PPV** | **NPV** | **MCC** |
| --- | --- | --- | --- | --- | --- | --- | --- | --- | --- | --- |
| MTR (0.74) | 6,965 | 3,300 | 31,510 | 16,578 | 0.66 | 0.30 | 0.91 | 0.68 | 0.66 | 0.26 |
| MTR (0.5) | 2,020 | 320 | 34,490 | 21,523 | 0.63 | 0.09 | 0.99 | 0.86 | 0.62 | 0.19 |
| MTR (0.25) | 475 | 52 | 34,758 | 23,068 | 0.60 | 0.02 | 0.99 | 0.90 | 0.60 | 0.01 |
| MPC (0.52) | 15,809 | 7,300 | 23,451 | 5,334 | 0.76 | 0.75 | 0.76 | 0.68 | 0.81 | 0.45 |
| MPC (2) | 3,047 | 414 | 18,096 | 30,337 | 0.64 | 0.14 | 0.99 | 0.88 | 0.63 | 0.23 |
| Polyphen-2 (0.908) | 14,280 | 4,762 | 25,989 | 6,863 | 0.76 | 0.68 | 0.85 | 0.75 | 0.79 | 0.47 |

Summary of the predictive performance of the MTR at cut-offs MTR < 0.74 (optimal cut-off for dataset), MTR < 0.5, MTR < 0.25 compared with the MPC at cut-offs MPC > 0.52 (optimal cut-off for dataset), MPC > 2, and compared with Polyphen-2 at Polyphen-2 > 0.908. Optimal cut-off values were defined using a recursive partitioning tree. TP, FP, TN and FN refer to the total true positives, false positives, true negatives and false negatives respectively. PPV, NPV and MCC refer to the positive predictive power (precision), negative predictive power and Matthew’s correlation coefficient respectively. Variants for each comparison are only reported where a valid score exists.

**Table S2 – Performance of MTR, MPC and Polyphen-2 for distinguishing between cancer-associated and neutral variants.**

| **Metric** | **TP** | **FP** | **TN** | **FN** | **Accuracy** | **Sensitivity** | **Specificity** | **PPV** | **NPV** | **MCC** |
| --- | --- | --- | --- | --- | --- | --- | --- | --- | --- | --- |
| MTR (0.68) | 2,911 | 4,378 | 59,208 | 9,857 | 0.81 | 0.23 | 0.93 | 0.40 | 0.86 | 0.19 |
| MTR (0.5) | 1,241 | 1,059 | 62,527 | 11,527 | 0.84 | 0.10 | 0.98 | 0.54 | 0.84 | 0.17 |
| MTR (0.25) | 269 | 210 | 63,376 | 12,499 | 0.83 | 0.02 | 0.99 | 0.56 | 0.83 | 0.08 |
| MPC (1.01) | 3,158 | 2,996 | 24,216 | 4,235 | 0.79 | 0.43 | 0.89 | 0.51 | 0.85 | 0.16 |
| MPC (2) | 978 | 609 | 26,603 | 6,415 | 0.80 | 0.13 | 0.98 | 0.62 | 0.81 | 0.10 |
| Polyphen-2 (0.908) | 3,308 | 4,438 | 22,774 | 4,085 | 0.75 | 0.45 | 0.84 | 0.43 | 0.85 | 0.13 |

Summary of the predictive performance of the MTR at cut-offs MTR < 0.68 (optimal cut-off for dataset), MTR < 0.5, MTR < 0.25 compared with the MPC at cut-offs MPC > 1.01 (optimal cut-off for dataset), MPC > 2, and compared with Polyphen-2 at Polyphen-2 > 0.908. Optimal cut-off values were defined using a recursive partitioning tree. TP, FP, TN and FN refer to the total true positives, false positives, true negatives and false negatives respectively. PPV, NPV and MCC refer to the positive predictive power (precision), negative predictive power and Matthew’s correlation coefficient respectively. Variants for each comparison are only reported where a valid score exists.


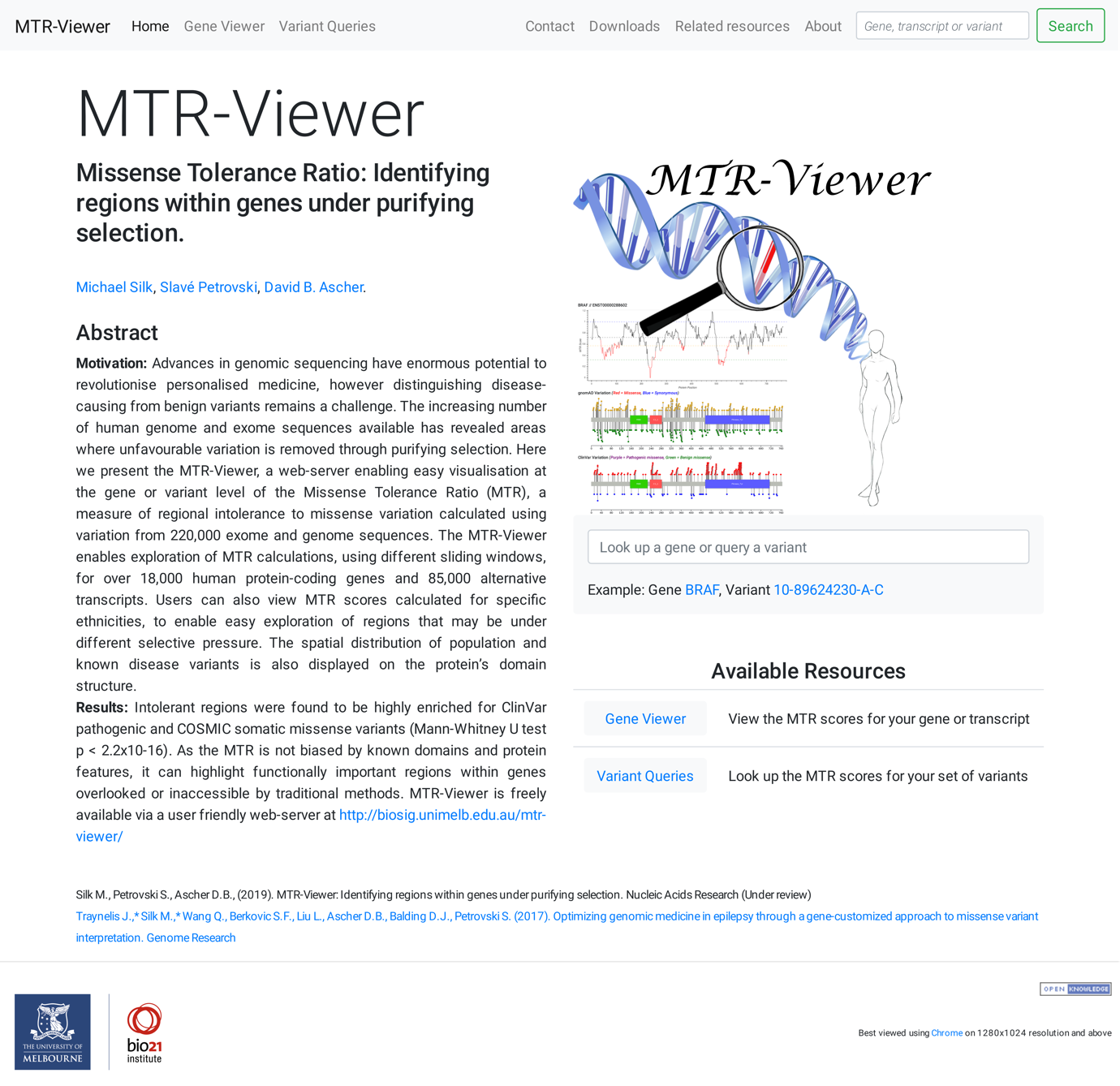


**Figure S1** – **MTR-Viewer home page.** Users can enter a gene or query a single variant here to be redirected to the appropriate page, or can select to run an example query.


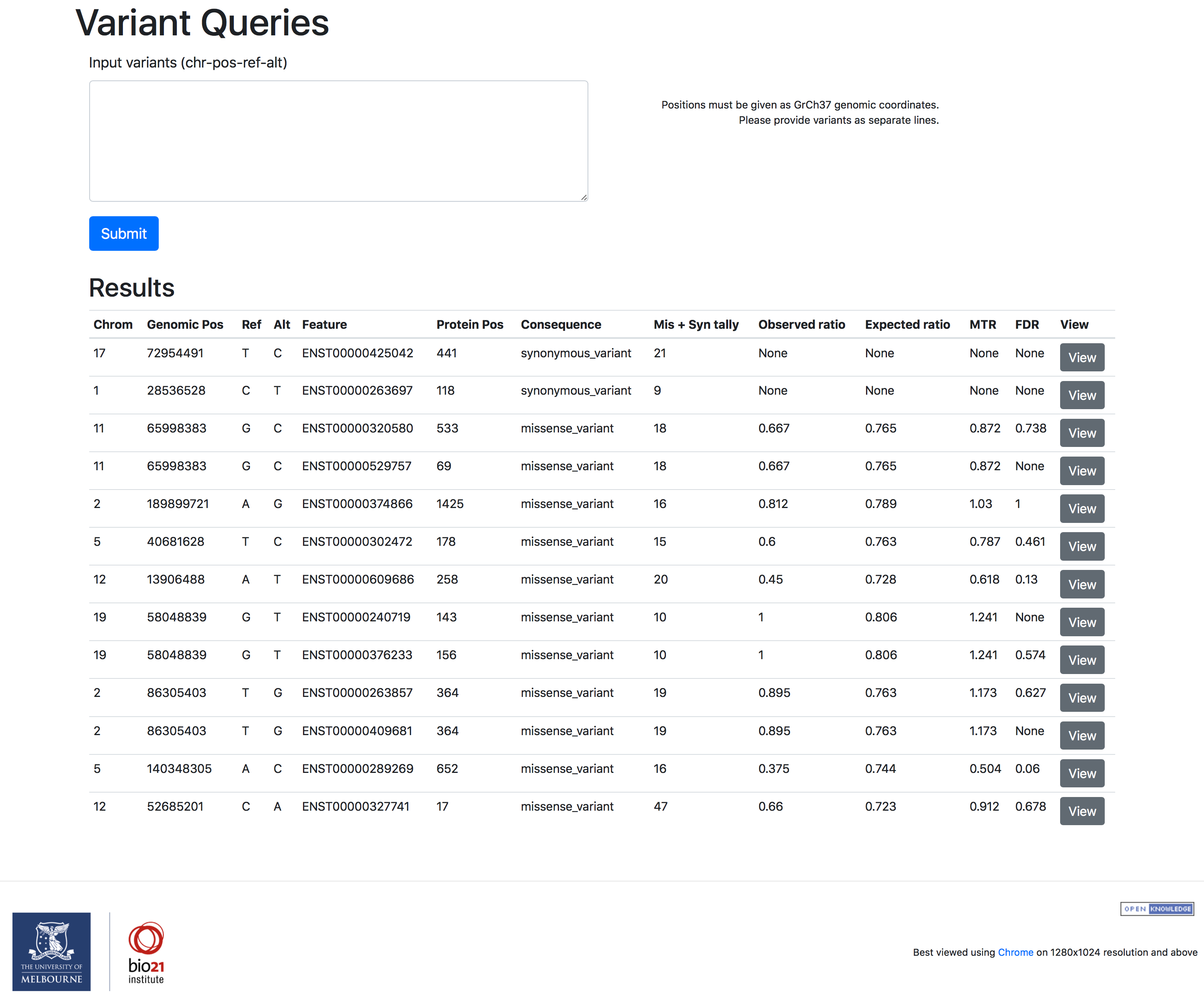


**Figure S2** – **Variant results page.** Results page for 10 example variants queried. For each variant, the web-server will show the MTR scores for all transcripts the variant is in. Non-matching variants are shown to the user.
